# ACTH Action on StAR Biology

**DOI:** 10.3389/fnins.2016.00547

**Published:** 2016-12-06

**Authors:** Barbara J. Clark

**Affiliations:** Department of Biochemistry and Molecular Genetics, University of LouisvilleLouisville, KY, USA

**Keywords:** StAR, adrenal, ACTH, translation, phosphorylation

## Abstract

Adrenocorticotropin hormone (ACTH) produced by the anterior pituitary stimulates glucocorticoid synthesis by the adrenal cortex. The first step in glucocorticoid synthesis is the delivery of cholesterol to the mitochondrial matrix where the first enzymatic reaction in the steroid hormone biosynthetic pathway occurs. A key response of adrenal cells to ACTH is activation of the cAMP-protein kinase A (PKA) signaling pathway. PKA activation results in an acute increase in expression and function of the Steroidogenic Acute Regulatory protein (StAR). StAR plays an essential role in steroidogenesis- it controls the hormone-dependent movement of cholesterol across the mitochondrial membranes. Currently StAR's mechanism of action remains a major unanswered question in the field. However, some insight may be gained from understanding the mechanism(s) controlling the PKA-dependent phosphorylation of StAR at S194/195 (mouse/human StAR), a modification that is required for function. This mini-review provides a background on StAR's biology with a focus on StAR phosphorylation. The model for StAR translation and phosphorylation at the outer mitochondrial membrane, the location for StAR function, is presented to highlight a unifying theme emerging from diverse studies.

## StAR (STARD1) discovery and function

The study of steroid hormone production has origins in work on adrenocorticotropin hormone (ACTH)-stimulated adrenal steroid synthesis (Macchi and Hechter, 1954a,b,c; Stone and Hechter, 1954). ACTH binds to its cognate 7-transmembrane G-protein coupled receptor, the melanocortin 2 receptor (encoded by the MC2R gene) that is located at the plasma membrane of adrenal fasciculata cells. ACTH binding to MC2R results in activation of multiple signal transduction pathways with the cAMP-dependent protein kinase A (cAMP-PKA) pathway being central to hormone-dependent activation of adrenal glucocorticoid and androgen synthesis (reviewed in Gallo-Payet and Payet, 2003; Spat et al., 2016). All steroid hormones are produced from cholesterol and the steroidogenic enzymes in cortisol synthesis, the major adrenal steroid produced in response to ACTH, have been reviewed in detail (Miller and Auchus, 2011). This mini-review focuses on the rapid or acute response to tropic hormonal stimulation- the movement of cholesterol into mitochondria. The first enzymatic reaction is the conversion of cholesterol to pregnenolone by the cytochrome P450 side chain cleavage enzyme (P450scc). P450scc is located in the mitochondrial matrix and work from the 1950s–1970s laid a solid foundation that cholesterol transport into mitochondria for access to P450scc was a key control point in steroidogenesis (Ferguson, 1962, 1963). The prevailing model at that time was that *de novo* synthesis of a protein factor upon hormonal stimulation was necessary for the cholesterol transfer. Furthermore, this factor needed to fulfill the following criteria: be newly synthesized upon hormonal stimulation in a time- and dose-dependent manner, be localized at the mitochondria, and have a short-half (reviewed in Clark and Stocco, 2014; Stocco et al., 2016). Here, I will highlight the studies which demonstrated that the Steroidogenic Acute Regulatory protein (StAR) fulfills the criteria for the acute regulator of steroidogenesis.

The first characterization of StAR was as phosphoproteins (pp with MW in kDa) pp37, pp32, and pp30 that appeared in rat adrenal after ACTH stimulation (Krueger and Orme-Johnson, 1983; Pon and Orme-Johnson, 1986; Pon et al., 1986; Alberta et al., 1989; Epstein and Orme-Johnson, 1991). Both *in vivo* and cell culture approaches provided strong correlative data that ACTH-cAMP-PKA induction of these proteins coincided with steroid production. In addition, the proteins were shown to be associated with mitochondria, and the pp32 and pp30 forms were processed forms of pp37 (Alberta et al., 1989; Epstein and Orme-Johnson, 1991; Krueger and Orme-Johnson, 1983; Pon and Orme-Johnson, 1986; Pon et al., 1986). Similar hormonal responsive protein(s) were characterized in the MA-10 mouse Leydig tumor cells, and ultimately the StAR protein was purified and cDNA cloned from this cell line (Clark et al., 1994). The deduced amino acid sequence encodes a protein with estimated molecular weight of 31.6 kDa with the amino-terminal region containing a classical mitochondrial targeting sequence (Clark et al., 1994). Expression of the cDNA in steroidogenic cells or *in vitro* in the presence of isolated mitochondria followed by Western blot analysis confirmed the cDNA encoded the pp37 protein previously characterized (Clark et al., 1994; King et al., 1995; Lin et al., 1995). The cDNA encoded a functional protein based on assays that measured increased steroid production in COS-1 cells or steroidogenic cells after transient expression of the cDNA. As anticipated, the 37 kDa StAR protein was imported and processed by mitochondria to generate the 30 kDa StAR protein (King et al., 1995). However, steroidogenesis ceases with removal of tropic hormone stimuli yet the 30 kDa form of StAR localized in the mitochondrial matrix is present with an estimated half-life of 4–5 h (Stocco and Sodeman, 1991; Granot et al., 2003). Thus, the requirement for a labile, short half-life criteria for the acute regulator of steroidogenesis required subsequent structure-function studies. Database searches using the cDNA and protein sequences revealed that StAR represented a novel protein (Clark et al., 1994). Shortly after the initial reports on StAR appeared, a conserved protein domain named the START domain (for steroidogenic acute regulatory protein (StAR)-related lipid-transfer domain), was identified using Web-based resources for predicting putative functional domains based on primary sequence data (Ponting and Aravind, 1999). Members of the START domain protein superfamily share a 210 amino acid region that folds into an α/β helix-grip fold structure containing a long hydrophobic cleft for lipid binding (reviewed in Stocco, 2001; Clark, 2012). The START domain within the StAR protein spans amino acids 65–285, which encodes the processed 30 kDa form, and binds cholesterol. Key studies showed that only the START domain is required for StAR's function: (1) addition of the 30 kDa StAR protein to isolated mitochondria promotes cholesterol transfer and pregnenolone production; and (2) expression of a cDNA encoding only the START domain, e.g., lacking the N-terminal mitochondrial targeting sequence (N62StAR), is capable of stimulating steroid production in steroidogenic cells or heterologous COS-1 cells (Arakane et al., 1996; Wang et al., 1998). Furthermore, mutations in the human *STAR* gene (*STARD1*) were identified in patients with congenital lipoid adrenal hyperplasia (lipoid CAH), a disorder marked by a lack of adrenal and gonadal steroidogenesis due to the inability to move cholesterol into the mitochondria [(Lin et al., 1995; Bose et al., 2000); reviewed in (Miller, 2014)]. The finding that *STAR* mutations are the genetic basis for lipoid CAH was key to establishing the essential role for StAR in ACTH-stimulated steroidogenesis as well as gonadotropin-stimulated steroidogenesis (Lin et al., 1995; Caron et al., 1997). A common mutation found in the *STAR* gene is a nonsense mutation, Q258X, which results in truncation of the last 28 amino acids (Nakae et al., 1997; Kim et al., 2011). Expression of C-terminal truncated forms of StAR in COS-1 cells confirmed that the loss of C-terminal helix, within the START domain, results in an inactive protein (Arakane et al., 1996; Wang et al., 1998).

The finding that N-terminal truncated StAR (N62StAR) was functional in cell-based assays, indicated that mitochondrial import was not required for function. However, the targeting of StAR to mitochondria may be important for efficient cholesterol transfer and steroid production *in vivo*. A mouse model of lipoid CAH was generated by “knocking out” StAR expression and, as anticipated, the animals lacked steroid production and significant amounts of cholesterol accumulated in the adrenals and gonads (Caron et al., 1997). Expression of a full-length *STAR* transgene in the StAR knockout mice restored adrenal and gonadal steroidogenesis while expression of the N62*STAR* transgene only partially restored steroidogenesis in a tissue- and gender-specific manner (Sasaki et al., 2008). The mice with the N62StAR transgene retained modest lipid accumulation in the adrenal and gonads. These data support that StAR is capable of functioning without the N-terminal mitochondrial targeting sequence, but highlight the importance of correct and efficient subcellular localization of the protein for full function *in vivo*. In the absence of mitochondrial import, N62StAR still associates with mitochondria outer membrane (Arakane et al., 1996; Wang et al., 1998).

## StAR phosphorylation and function

Early studies proposed an association between StAR phosphorylation and function (Pon et al., 1986; Chaudhary and Stocco, 1991; Clark et al., 1995; Hartigan et al., 1995). A PKA-dependent phosphorylation at S194 (mouse) or S195 (human) was validated as an important post-translational modification required for StAR's function in cell-based and *in vitro* assays (Arakane et al., 1996; Jo et al., 2005; Baker et al., 2007). StAR-S194/195 nomenclature reflects the deletion of 3 nucleotides in exon 2 that eliminates a residue which lies within the cleavable mitochondrial signal sequence in mouse StAR. A key study confirmed the relevance for StAR-S194 phosphorylation *in vivo*; StAR knock-out mice were used to demonstrate that re-expression of wild-type StAR but not StAR-S194A restored steroidogenesis in the adrenal and testis (Sasaki et al., 2014). The newborn StAR-S194A transgenic male mice had lipid accumulation in the adrenal and testis, and circulating hormone levels (high ACTH and low corticosterone and testosterone) similar to StAR knock-out mice. Thus, StAR phosphorylation at S194/195 is critical for function *in vivo*.

The molecular mechanism of action for StAR-mediated cholesterol transport across the mitochondrial membranes is not fully defined, therefore it remains a challenge to define the role of S194/195 phosphorylation in StAR's function. The current model for StAR-mediated cholesterol transport asserts that protein-protein interactions between StAR and OMM protein(s) triggers cholesterol transfer (Bose et al., 1999; Mathieu et al., 2002; Baker et al., 2007; Roostaee et al., 2008; Rajapaksha et al., 2013). Structural changes in the START domain induced by cholesterol binding and by interaction with the OMM as well as the kinetics of cholesterol transfer across the mitochondrial membranes have been reviewed elsewhere (Miller, 2007). Precursor StAR processing to the mitochondrial 30 kDa form is reported to be very efficient, stoichiometric, and dependent upon cholesterol-induced structural changes (Artemenko et al., 2001; Rajapaksha et al., 2013). Import of StAR is proposed to be the “off switch” for cholesterol transfer due to loss of StAR interaction with an OMM protein complex (Bose et al., 1999; Miller, 2007). However, the transit time at the OMM contributes to StAR function with slower mitochondrial import associated with greater activity (Bose et al., 2002). Does StAR phosphorylation affect cholesterol binding or mitochondrial import? Purified hStAR START domain containing the S195A mutation was shown to have the same cholesterol binding kinetics as wild-type StAR START domain, indicating that phosphorylation is not required for cholesterol binding (Baker et al., 2007). StAR-S194A/S195A or non-phosphorylated forms of wild-type StAR are efficiently processed to the 30 kDa mitochondrial matrix form both in cell culture and *in vivo* studies, thus phosphorylation doesn't appear necessary for StAR import (Arakane et al., 1997; Jo et al., 2005; Sasaki et al., 2014; Clark and Hudson, 2015). The region that slows StAR import is a protease-resistant domain that spans amino acids 63–188 (Bose et al., 1999, 2002), therefore, it is unlikely that S194/195 phosphorylation alters StAR import rates, although this has not been directly tested.

While StAR phosphorylation doesn't appear to be required for mitochondrial import, understanding the import mechanism may provide insight into StAR-OMM interactions that are required for function. TOM20 and TOM22 are the receptor components of the Translocase of the outer membrane (TOM) complex that function with the pore-forming TOM40 complex to control protein import into mitochondria (Becker et al., 2012; Harbauer Angelika et al., 2014). Classically, post-translational protein import via a TOM20/22-TOM40 pathway is associated with proteins that are synthesized in the cytoplasm with a cleavable amino-terminal amphipathic helix that serves as a mitochondrial matrix targeting sequence, such as StAR (reviewed in Harbauer Angelika et al., 2014, Figure 1). The proteins are in complex with chaperones to maintain an unfolded state for import while the amino terminal sequence binds TOM20/22 receptors (Harbauer Angelika et al., 2014). However, a co-translational mechanism for import of matrix-localized proteins with amino-terminal cleavable signal sequences was proposed over 40 years ago (Kellems et al., 1974) and recent data in yeast support this model. mRNAs co-purify with isolated mitochondria in wild-type yeast strains but not TOM20 deficient strains following cycloheximide (CHX) treatment (Eliyahu et al., 2010). These data indicate that blocking translation and stabilizing polysomes by CHX enhances ribosome-mRNA interactions with mitochondria in a TOM20-dependent manner. StAR has been shown to co-purify with TOM22 and siRNA knock-down of TOM22 in MA-10 cells results in diminished cholesterol metabolism by isolated mitochondria (Prasad et al., 2015, 2016). Since the StAR import mechanism is incompletely described, I use TOM20/22 to reflect both receptors work together for preprotein import in the classical mitochondrial import model. However, it may be that TOM20 and TOM22 can serve redundant functions for StAR import while TOM22 is required for StAR function (Rajapaksha et al., 2013, 2016).

**Figure 1:**
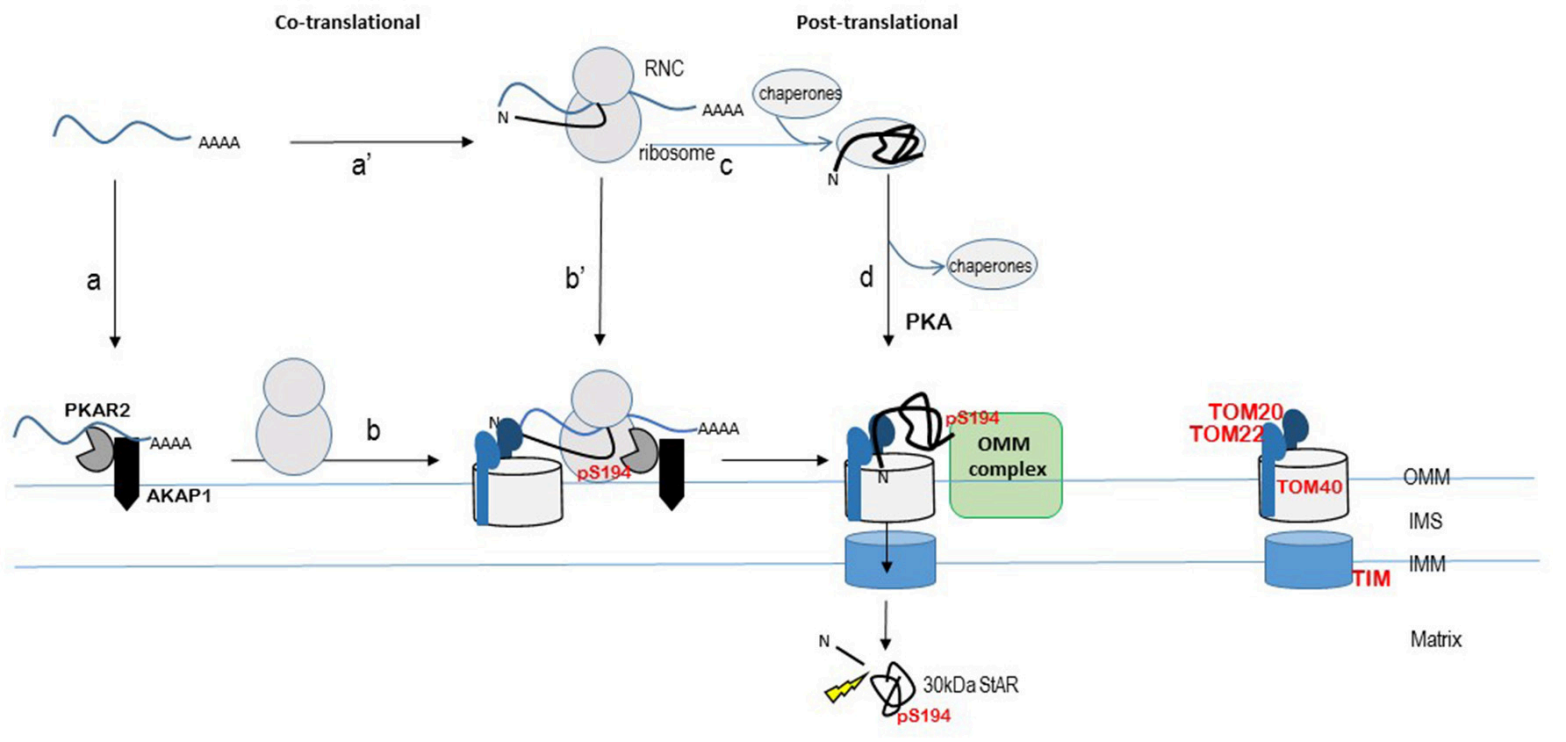
**Model for localized StAR translation and phosphorylation at mitochondria**. The TOM20/22-40 complex is an established pathway for import of matrix-localized proteins that contain an N-terminal cleavable signal peptide. StAR is modeled within this import pathway, although the import pathway for StAR remains to be determined. The ribosome nascent chain complex (RNC) is shown as StAR mRNA bound by the ribosome and protein depicted as black line with the N-terminus denoted (N). The START domain is depicted by the “folded” black line. StAR translation is modeled on both free ribosomes (steps c and d) and mitochondria-associated ribosomes (steps a to b or a' to b') as described in the text. Cleavage of the N-terminal signal sequence by matrix metalloproteases (indicated by the thunderbolt) produces the 30 kDa STAR protein (START domain) in the matrix. The import of StAR is the “off switch” for cholesterol transfer due to loss of interaction with an OMM protein complex. The OMM protein complex (indicated here as a green rectangle) that facilitates the StAR-dependent cholesterol transfer from the OMM to the matrix remains to be determined. Proposed OMM components of this complex include StAR, AKAP, PKA, VDAC1/2, TOM22, PCP, TSPO, and ACBD3. The OMM protein that interacts specifically with phosphoStAR to trigger complex formation for cholesterol transfer across the mitochondrial membranes remains to be identified. TIM (Translocase of the inner membrane) complex; IMS, intermembrane space; OMM, outer mitochondrial membrane; IMM, inner mitochondrial membrane. Concepts of this model have been presented by others in Dyson et al. (2008), Poderoso et al. (2009), Grozdanov and Stocco (2012), Aghazadeh et al. (2015), Prasad et al. (2015), Lee et al. (2016b), Midzak and Papadopoulos (2016), and Paz et al. (2016).

Localized translation and post-translational modification of StAR at the mitochondria is an attractive model for the acute control of steroidogenesis (Dyson et al., 2008; Eliyahu et al., 2010; Lesnik et al., 2015). This model is supported by data that show StAR mRNA was associated with mitochondrial A-kinase anchoring protein 121 (AKAP121) and type II PKA in MA-10 mouse Leydig cells (Dyson et al., 2008, 2009). Independent studies have also demonstrated a PKA-dependent co-localization of AKAP121, acyl-CoA, and upstream ERK1/2 kinases with StAR at the mitochondria (reviewed in Poderoso et al., 2009; Paz et al., 2016). Thus, the co-localization of PKA and AKAP proteins at the mitochondria is emerging as a consistent theme. siRNA-mediated silencing of AKAP121 in MA-10 Leydig cells resulted in loss of PKA (PKAR2) association with mitochondria, independent of Bt_2_cAMP treatment, indicating that an AKAP121-PKAR2 complex is constitutively poised at the OMM (Dyson et al., 2008). Immunofluorescence-based detection of AKAP1 in H295R human adrenocortical cell line showed co-localization with TOM20, indicating localization with mitochondria in an adrenal cell line (Grozdanov and Stocco, 2012). Furthermore, a portion of StAR 3′UTR mRNA was shown to directly bind to AKAP1 and in H295R cells and StAR mRNA was recovered following AKAP1 immunoprecipitation (Grozdanov and Stocco, 2012). The StAR mRNA-AKAP1 interaction was detected only after Bt_2_cAMP treatment of H295R cells suggesting that newly transcribed StAR mRNA associates with AKAP1. Our earlier work demonstrated that StAR mRNA levels and endogenous protein levels are greatly diminished in the PKA deficient mouse Kin-8 adrenocortical cells following Bt_2_cAMP treatment compared to the PKA responsive Y1 adrenocortical cells (Clark et al., 1997; Clark and Hudson, 2015). Yet despite the lower StAR mRNA levels, the mRNA was associated with actively transcribing ribosomes and StAR protein synthesis rates were the same in both cell lines, indicating translation is intact in the absence of PKA signaling (Clark and Hudson, 2015; Clark et al., 1997). Conversely, in MA-10 cells that lack AKAP1 via siRNA-mediated silencing, Bt_2_cAMP treatment increased StAR steady-state mRNA levels but StAR protein levels were diminished. Together the data support that newly transcribed StAR mRNA associates with AKAP1 in adrenocortical cells (Kin8 and H295R cells) and that the AKAP1-StAR mRNA association contributes to translation efficiency. The model that AKAP1 localizes PKAR2 at the OMM provides a platform for localized phosphorylation of StAR (Grozdanov and Stocco, 2012; Merrill and Strack, 2014, Figure 1). Recently StAR mRNA association with mitochondria in MA-10 cells was demonstrated using high resolution fluorescence *in situ* hybridization (Lee et al., 2016a). Key findings from this work indicate that StAR mRNA-mitochondrial interactions are dynamic, and suggest that when StAR transcription is maximally activated, newly transcribed and processed StAR mRNA is delivered to the cytoplasm and mitochondria for efficient translation.

The prevailing concept is that StAR phosphorylation influences key interactions with OMM proteins that promote cholesterol transfer. The challenge is that several StAR-interacting complexes at the OMM have been proposed yet the identity of the functional StAR cholesterol transfer complex remains to be validated (Bose et al., 2008; Rone et al., 2012; Issop et al., 2015). Early work characterized a OMM protein complex that included 18 kDa translocator protein (TSPO) and voltage-dependent anion channels 1 (VDAC1) (Liu et al., 2006). The working model was that upon hormonal stimulation StAR is expressed and associates with acyl-coenzyme A binding domain containing 3 protein (ACBD3), previously referred to as TSPO-associated protein (PAP7), leading to a StAR-PKA-ACBD3-TSPO-VDAC1 complex at the OMM (reviewed in Papadopoulos et al., 2015; Midzak and Papadopoulos, 2016). *In vitro* studies using mitochondria isolated from sheep adrenal incubated with radiolabeled, *in vitro* synthesized StAR protein identified VDAC1 and phosphate carrier protein (PCP, SLC25A3) as components of a StAR-containing complex (Bose et al., 2008). VDAC1, VDAC2, TOM22, and StAR have been shown to be part of large multiprotein complexes purified from mitochondria or mitochondria-associated endoplasmic reticulum (ER) membrane (MAM) regions isolated from rat testes and MA-10 mouse Leydig tumor cells (Prasad et al., 2015). The functional significance for these proposed complexes is based on data that demonstrated siRNA-mediated silencing and/or pharmacological inhibition of TSPO, VDAC1, VDAC2, or ACBD3 decreased or blocked cAMP-PKA-mediated steroid production in steroidogenic cells or StAR-mediated steroid production in COS-1 cells. However, mouse models lacking either TSPO or VDAC1 expression are viable and fertile (Weeber et al., 2002; Sileikyte et al., 2014; Tu et al., 2014), although VDAC1^−/−^ mice present with mitochondrial alterations in a strain-dependent manner. Loss of VDAC2 results in embryonic lethality (Anflous et al., 2001; Craigen and Graham, 2008). These studies indicate that TSPO and VDAC1 are not obligatory for steroidogenesis, and suggest a redundancy of function for cholesterol transfer may occur in the absence of these factors. The question is whether StAR phosphorylation drives interaction with any of these OMM proteins. siRNA-mediated silencing of VDAC1 or PCP protein expression in COS-1 cells resulted in decreased vector-driven StAR protein levels with a corresponding decrease in phosphoStAR levels and steroidogenic response (Bose et al., 2008). The 30 kDa StAR was detected by Western blot analysis indicating that mitochondrial import of StAR was intact in VDAC1 null cells. However, 30 kDa phosphoStAR was greatly diminished indicating that in the absence of VDAC1, import of phosphorylated StAR into mitochondria was blocked. Addition of cysteine protease inhibitors to the VDAC1 deficient cells restored StAR phosphorylation and phosphoStAR mitochondrial import and steroid production to levels observed in control cells (Bose et al., 2008). One interpretation of these data is that VDAC1 may help stabilize precursor StAR allowing for StAR phosphorylation and enhanced cholesterol transport function. In a separate study, COS-1 cells with siRNA-mediated loss of VDAC2 expression retained vector-driven StAR synthesis and phosphorylation but there was no steroidogenic response (Prasad et al., 2015). In the absence of VDAC2, as with VDAC1, no mitochondrial import of phosphoStAR was detected (unphosphorylated StAR was not measured) (Prasad et al., 2015). Furthermore, a StAR-VDAC2 complex was immunoprecipitated from COS-1 cells overexpressing VDAC2 and N62StAR, providing the first evidence for a possible direct interaction between StAR and an OMM protein (Prasad et al., 2015). These data suggest that synthesis of phosphorylated precursor StAR is not sufficient for function, and support a role for specific phosphoStAR-OMM interactions mediating cholesterol transfer. The authors propose that StAR is synthesized at MAM regions and interacts with VDAC2 for subsequent mitochondrial import and StAR processing (Prasad et al., 2015). Perhaps the loss of phosphoStAR import in the absence of either VDAC1 or VDAC2 reflects a loss of StAR's N-terminal interaction with the TOM20/22-TOM40 complex, leading to loss of StAR structure which is necessary for cholesterol transfer function of StAR (Rajapaksha et al., 2013; Prasad et al., 2015, Figure 1).

In summary, ACTH action on StAR biology requires an immediate response to hormone stimulation, which is seen in a rapid increase in StAR transcription. StAR translation, phosphorylation, and mitochondrial import are likely coordinated processes, but the mechanisms regulating these steps are lacking. Nevertheless, the work over the past decade supports a model for localized translation at the mitochondria. One proposed scenario is the newly transcribed StAR mRNA binds AKAP1 through 3′UTR-driven interactions localizing the mRNA to the mitochondria where it is bound by ribosomes and translated (Figure 1, step a and b). Alternatively, cytoplasmic StAR mRNA is bound by ribosomes and the ribosome-nascent-chain is directed to mitochondria where the interaction is stabilized by 3′UTR mRNA-AKAP1 interaction, similar to co-translational models described for yeast (Figure 1, step a' and b'). StAR mRNA 3′UTR driving mitochondrial location and localized translation could explain the amino-terminal truncated form of StAR (N62StAR), which lacks the signal sequence, being localized to the OMM and functional. Translation of the amino-terminal mitochondrial signal sequence, on the other hand, could lead to recognition by the TOM20/22-TOM40 complex, thereby tethering StAR to the TOM complex. StAR translation continues and phosphorylation of S194/195 of the START domain occurs in a co-translational process by the mitochondrial localized PKA. Post-translational mitochondrial targeting of StAR would follow the classical pathway; StAR is synthesized on cytoplasmic polysomes, bound by chaperones, and targeted to TOM20/22 by the N-terminal signal sequence (Figure 1, steps c and d). StAR phosphorylation could occur at any step in this classical pathway. In both the co- and post-translational models, there must be a mechanism allowing for folding of the START domain and phosphorylation of S194/195 that are necessary for interactions at the OMM that promote cholesterol transfer. StAR import is slowed by a pause domain (Bose et al., 1999, 2002), and this could permit time necessary to generate phosphoStAR by mitochondrial localized PKA. The retention of phosphoStAR's START domain at the OMM signals for cholesterol transfer. Thus, studies directed at testing StARS194/195-OMM protein interactions may help uncover the ACTH-dependent phosphoStAR-OMM protein interaction specific for cholesterol transfer.

## Author contributions

BC reviewed the literature, generated the figure, and wrote the mini-review.

### Conflict of interest statement

The author declares that the research was conducted in the absence of any commercial or financial relationships that could be construed as a potential conflict of interest.
